# Multiple nodules in the scrotal wall

**DOI:** 10.1002/ccr3.5388

**Published:** 2022-02-07

**Authors:** Chaima Kouki, Mariem Amouri, Slim Charfi, Tahya Boudawara, Hamida Turki

**Affiliations:** ^1^ Department of Dermatology Hedi Chaker Hospital Sfax Tunisia; ^2^ Department of Anatomopathology Habib Bourguiba Hospital Sfax Tunisia

**Keywords:** calcifying nodule, idiopathic diseases, scrotal calcinosis

## Abstract

Herein, we report a rare case of extensive ISC. It affects dark‐colored skin men aged from 20 to 40 years old, suggesting an ethnic susceptibility. Pathogenesis of scrotal calcinosis remains controversial.

## Graphical abstract

Despite idiopathic scrotal calcinosis (ISC) is a common pathology, extensive forms remain rare. The aesthetic discomfort represents the main complaint and may severely affect sexual life. Herein, we report a rare case of extensive ISC.

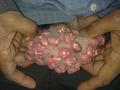

## 1 PRESENTATION

**FIGURE 1:**
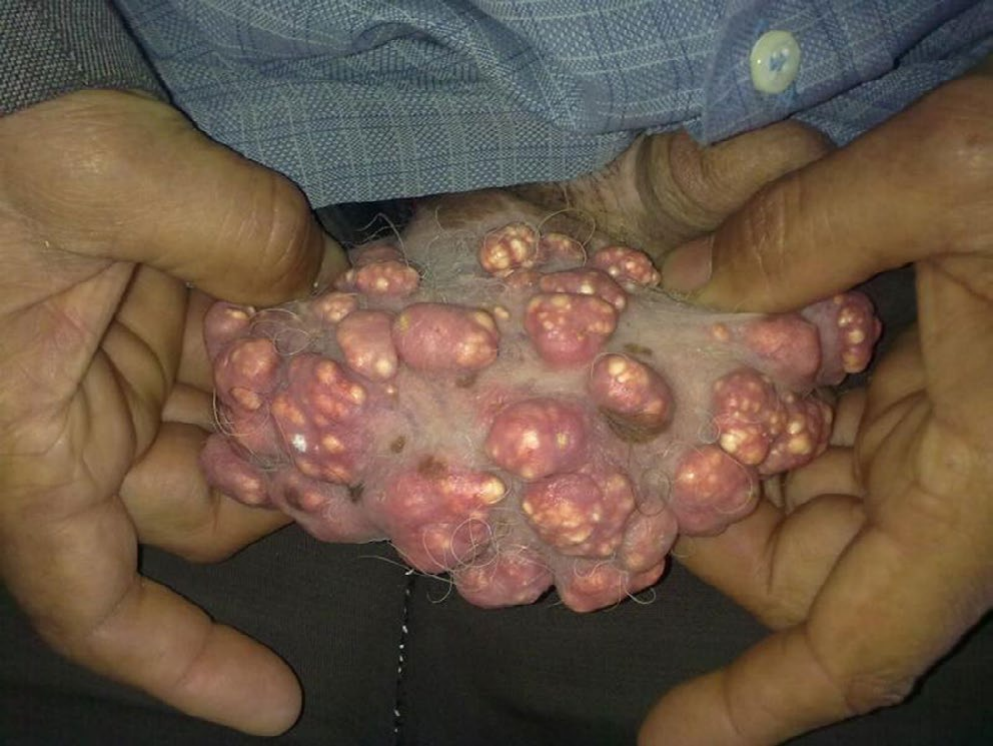
Multiple yellowish firm nodules on the scrotal wall

**FIGURE 2:**
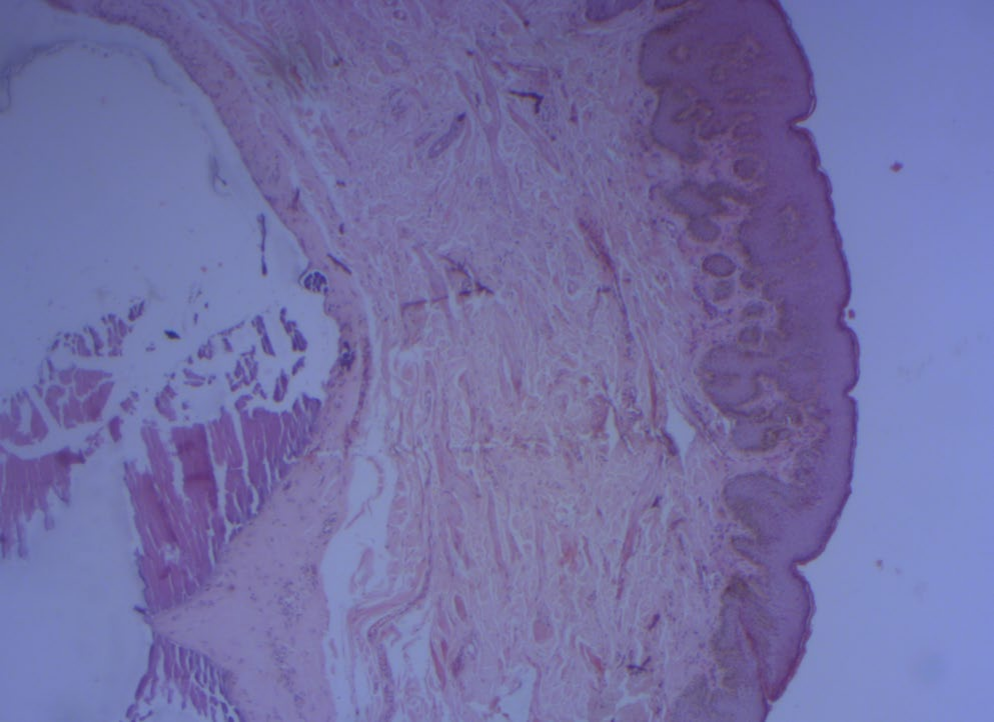
Large globular basophilic deposits of calcium within the dermis (HE ×25)

A 52‐year‐old man with no significant medical history presented to our department with multiple scrotal nodules. The lesions had gradually increased in number and size during 2 decades. Physical examination (Figure 1) revealed prominent 2–20 mm yellowish firm papules or nodules on the scrotum. There were no areas of ulceration or discharge. Laboratory examinations (serum calcium, phosphorus, thyroid hormone, and alkaline phosphatase levels) were normal. Histological examination of an excised nodule revealed intradermal deposition of calcium surrounded by histiocytes without cystic structure (Figure 2).

The diagnosis of idiopathic scrotal calcinosis (ISC) was retained, and the patient was referred to the surgical department for wide excision.

Herein, we report a rare case of extensive ISC. It affects dark‐colored skin men aged from 20–40 years old, suggesting an ethnic susceptibility.^1^ Pathogenesis of scrotal calcinosis remains controversial. Histopathologic examination reveals deposition of calcium in the dermis surrounded by histiocytes, macrophages, and lymphocytic infiltrates.^1^

There are no associations with systemic comorbidities.^1^ Surgery is the most common treatment approach.^2^ Ablative lasers (YAG, CO2 Laser) have been also proposed. Although the possibility of ISC recurrence after surgery has been debated, only few cases of ISC recurrence were reported in the literature.^2^

## CONFLICTS OF INTERESTS

We have no conflict of interest to disclose concerning this work.

## AUTHOR CONTRIBUTION

Dr. Chaima Kouki involved in writing the manuscript and submitting the article. Pr Mariem Amouri contributed to the design and wrote the manuscript. Pr Charfi Slim involved in providing and writing the anatomopathology part of the manuscript. Pr Turki Hamida and Boudawara Tahya supervised and approved the final manuscript. All authors discussed the results and commented on the manuscript.

## CONSENT

The authors certify that they have taken necessary consent from patient for publication of images.

## Data Availability

No data are available.
